# Calculation of Dynamic Viscosity in Concentrated Cementitious Suspensions: Probabilistic Approximation and Bayesian Analysis

**DOI:** 10.3390/ma14081971

**Published:** 2021-04-14

**Authors:** Ángel De La Rosa, Gonzalo Ruiz, Enrique Castillo, Rodrigo Moreno

**Affiliations:** 1 ETS de Ingenieros de Caminos, C. y P., Universidad de Castilla-La Mancha, Av. Camilo José Cela s/n, 13071 Ciudad Real, Spain; Angel.delaRosa@uclm.es; 2 Real Academia de Ingeniería, Don Pedro 10, 28005 Madrid, Spain; castie@unican.es; 3 Instituto de Cerámica y Vidrio (CSIC), C. Kelsen 5, Campus de Cantoblanco, 28049 Madrid, Spain; rmoreno@icv.csic.es

**Keywords:** cementitious suspensions, viscosity, Krieger–Dougherty equation, deterministic and probabilistic models, Bayesian analysis

## Abstract

We present a new focus for the Krieger–Dougherty equation from a probabilistic point of view. This equation allows the calculation of dynamic viscosity in suspensions of various types, like cement paste and self-compacting mortar/concrete. The physical meaning of the parameters that intervene in the equation (maximum packing fraction of particles and intrinsic viscosity), together with the random nature associated with these systems, make the application of the Bayesian analysis desirable. This analysis permits the transformation of parametric-deterministic models into parametric-probabilistic models, which improves and enriches their results. The initial limitations of the Bayesian methods, due to their complexity, have been overcome by numerical methods (Markov Chain Monte Carlo and Gibbs Sampling) and the development of specific software (OpenBUGS). Here we use it to compute the probability density functions that intervene in the Krieger–Dougherty equation applied to the calculation of viscosity in several cement pastes, self-compacting mortars, and self-compacting concretes. The dynamic viscosity calculations made with the Bayesian distributions are significantly better than those made with the theoretical values.

## 1. Introduction

The rheological behavior of a cement paste depends principally on the contents of solid particles and their capacity to form flocs as a consequence of the particle interactions, which can be modulated by adding adequate dispersing agents to improve the dispersion state. As the particle contents increases, the value of dynamic viscosity, $\eta_{p}$, also increases [1]. If these systems are considered homogenous suspensions, the Krieger–Dougherty equation [1,2] gives the dynamic viscosity from the volume fraction of cement particles, which may be obtained from the water/cementitious materials relationship, w/cm [3]. The interest and application of the Krieger–Dougherty equation in these suspensions are due, from a theoretical point of view, to its robustness and the fact that its parameters have a physical significance [1,4,5,6]. The Krieger–Dougherty equation [1,2,6] is defined as follows:

(1) $$\frac{\eta }{\eta_{0}}={\left(1\;-\;\frac{\phi }{\phi_{m}}\right)}^{-\left[\eta \right]\;\phi_{m}}$$

where,

η: dynamic viscosity of the suspension.
$\eta_{0}$: dynamic viscosity of the fluid phase of the suspension.
ϕ: fraction in volume of the disperse solid phase of the suspension.
$\phi_{m}$: maximum packing fraction of particles in the disperse solid phase.
[η]: intrinsic viscosity, which depends on the specific viscosity (ratio between the viscosity of the suspension and the dispersion liquid) and the volume fraction of solids.

Much research has been performed on the rheology of the various types of cement suspensions in which the suitability of this equation for the calculation of dynamic viscosity has been verified [2,4,5,7,8]. In addition, a comparison has been made of the values calculated with the experimental measures performed with rheometers, thereby obtaining good results with cement pastes with and without superplasticizer admixtures [1,3]. The Krieger–Dougherty equation [1,2,6] depends on two parameters: the maximum packing fraction, $\phi_{m}$, and the intrinsic viscosity, [η]. The first is a measurement of the maximum packing that may be reached in a particle system, i.e., the maximum concentration of particles that can be added while maintaining some flowability. It depends on the dispersion conditions, on the shape of the particles and, especially, on their distribution and size [3,6]. The second measures the individual effect of the particles on the viscosity and is secondary to their form [1]. Moreover, both parameters are affected by the shear rate, $\dot{\gamma }$, applied in the system: as $\dot{\gamma }$ increases, $\phi_{m}$ tends to increase while [η] shows the opposite effect [6]. Nevertheless, it is certain that the behavior of [η] is very dependent on the slenderness of the particles [1,9].

Generally, in cement suspensions both $\phi_{m}$ as well as [η] may be determined from the adjustment of a collection of experimental measurements, thereby assuming the hypothesis of sphericity and rigidity of the particles [10,11,12] and the formation of monodisperse or polydisperse systems that adapt geometric packing of known values (cubic, random, or hexagonal compact packing) [7]. In such cases, it is common practice to consider values ranging between 0.6–0.7 for $\phi_{m}$, and equal to 2.5 for [η] (in monodisperse systems), and greater than 0.7 for $\phi_{m}$, and equal to 2.5 for [η] when polydispersion increases [1,13]. However, as the asymmetry of the particles increases, $\phi_{m}$ may reach values below 0.6, [η] between 3 and 5 when the particles are sharp and approximately equal, and between 4 and 10 in particles with more acicular shapes [1,13]. Also, the trend in the behavior of both parameters when shearing the system suggests that the product of the two, which appears in the exponent of the Krieger–Dougherty equation, remains practically constant in any case [7,8]. All the mentioned values for the parameters follow from the hypothesis on the shape and type of arrangement of the particles, rather than from experiments. Besides, the variations of these parameters due to the random shape and arrangement of the solid phases are not known, which makes the application of the Bayesian analysis to know their probability distributions desirable.

Since a physical system is not deterministic, but random, the models that simulate its behavior should be probabilistic, if a good approximation to reality is sought. Therefore, the application of parametric-deterministic models should be supplemented with parametric-probabilistic models, which signifies added value within the field of modeling in engineering practice. For this reason, it is adequate to identify the sources of randomness that are associated with these systems, observe them, and perform trials and collect data to establish processes for the construction of probabilistic models [14,15]. The transformation of parametric-deterministic models into parametric-probabilistic models is usually performed using Bayesian analysis methods. Due to their complexity, these methods were limited a short time ago to the conjugate family of functions, which were the only ones for which expressions of posterior distributions functions could be easily determined. Nevertheless, the appearance of Markov Chain Monte Carlo methods and the Gibbs Sampling has enabled the simulation of the posterior distribution directly, thereby generalizing its application [16,17,18,19].

Within the scope of the study of the rheology of systems that are based on cement materials, and more specifically, on suspensions of cement paste, the colloidal nature of the particles that intervene, and the physical–chemical interactions that are adjusted as a consequence of the inclusion of admixtures, make these suspensions especially attractive for the rheology study from a probabilistic point of view, and they may be analyzed through Bayesian methods. In the same manner, more complex cement systems, like self-compacting mortar and self-compacting concrete, show the same tendency insofar as the relationship between viscosity and particle content, even with some differences associated with the presence of larger size solids, such as aggregates.

These analyses are of great engineering relevance [20], since self-compactibility, passing ability, pumpability, etc. are properties that depend on the viscosity and, in general, on the rheological properties of the mixture. In turn, these properties depend on the composition of the suspension, particularly water (its hardness and contents, especially metallic ions), and the geometry and compactness of cementitious and aggregate materials in suspension. For these reasons, the principal objective of this research consists of the transformation of the Krieger–Dougherty equation into a parametric-probabilistic model using Bayesian analysis, and to apply it to cement pastes, self-compacting mortars, and self-compacting concretes. We also want to use the new model to compute the probability density functions that intervene in the Krieger–Dougherty equation applied to these cementitious suspensions.

The article is structured as follows: Firstly, we define the characteristics of the Bayesian analysis and its suitability for application to the Krieger–Dougherty equation. Next, the paper describes the procedure performed with the methodology. Subsequently, there is a description of the experimental campaign undertaken and the scientific literature data used, which is followed by the results and its discussion. Lastly, the conclusions drawn from the research are explained.

## 2. Probabilistic Model and Bayesian Analysis of the Krieger–Dougherty Equation

The models of probabilistic networks are an appropriate methodology for dealing with problems in the engineering practice [15] since the reality is random, not deterministic. When we deal with multivariate random variables, our aim is to obtain the multivariate density or probability function, because if these functions are known we can answer any probability question about this variable, for example, the marginal densities of any subset of variables, the regression expressions, any conditional distributions, etc. However, the definition of a multivariate random variable is neither trivial nor easy. Some definitions do not guarantee the existence of multivariate distributions. For example, the conditionals of *x* given *y*, and *y* given *x* can be incompatible. The best way of defining multivariate distributions is by means of a Bayesian network, because they always guarantee the existence of the joint multivariate density and, in addition, the definition of this density is made locally, that is, in small pieces that always guarantee this existence.

The objective of the equation of Krieger–Dougherty [2] (Equation (1)) is the analytical calculation and prediction of the viscosity as a function of the volume fraction of solids in suspensions of different types. Within systems based on cement materials, this equation has been used to calculate the viscosity of cement pastes [1] and self-compacting concrete [7]. In dealing with an equation in which two parameters ($\phi_{m}$ and [η]) intervene, they may be adjusted to the experimental data [10], which exhibits variety in the values that they may acquire and that depend on the characteristics of the suspensions. The physical-chemical nature of the materials and the interactions that are produced among them, inherently exhibit a collection of responses of a random nature. Thus, the parameters controlling such responses may be deemed random variables that follow density functions of probability, in lieu of being defined with a single value.

Therefore, the equation of Krieger–Dougherty may be expressed in probabilistic terms, thereby obtaining information on the variability of the estimation of η. If the matter is dealt with by using a classic methodology, η may be treated as a random variable of a parametric family, thereby reducing the problem to an estimation of equation parameters. Nevertheless, if a Bayesian methodology is applied, a series of parametric distribution families are considered, and their parameters are treated as random variables [21].

### 2.1. Sources of Randomness in Cementitious Suspensions

A colloidal suspension is a system of two or more phases formed by a fluid dispersing medium and dispersed particles with diameters between $10^{-9}$ and $10^{-5}$ m [22,23]. They vary from large molecules, such as polymer chains of superplasticizer admixture, to small particles, such as cement materials and mineral fillers [22,24]. The shape and the size distribution of the particles, together with the surface chemistry and the interaction forces among them and with the dispersing medium, determine the properties of the suspension [22]. The forces of interaction (van der Waals forces, electrostatic repulsion forces, steric hindrance, and Brownian forces) dominate on the inertials and the gravitationals at this scale [22]. This constitutes one of the sources of randomness in suspensions of cement paste together with the random nature itself of the Brownian movement [1,9]. Another of the sources arises from the variety of shapes (pseudo-spherical, angular, elongated etc.) and sizes of the particles of the cement materials [1,25]. The shear rate, $\dot{\gamma }$, to which the system is subjected must also be taken into account. It has a direct influence on the packing and interaction among the particles, which is related to the values that the parameters $\phi_{m}$ and [η] adopt [1,6,7,8].

Regarding self-compacting mortar (SCM) and self-compacting concrete (SCC), they may be deemed as systems composed of a solid granular phase of one or various sizes, respectively, that is found in suspension in a continuous fluid viscous phase, such as cement paste [6,7,26]. The cement paste has a pronounced random nature as has been explained before. The rheological behavior of SCM and SCC is conditioned by the interactions between the aggregates and by the physical–chemical properties of the cement paste [26]. Thus, performing a multi-scale approach seems appropriate since the different phases are defined by the maximum size of their particles [26], and each exhibits a characteristic rheology.

If the hypothesis of considering SCM and SCC granular skeleton as a collection of rigid non-colloidal polydisperse spheres is proposed with respect to the distribution of the size of the particles, the viscosity of this system may be determined using the Krieger–Dougherty equation, Equation (1). As the fluid phase is always water (and thus $\eta_{0}$ is not supposed to vary much), Equation (1) depends mainly on two parameters with physical significance: the maximum packing fraction of the particles, $\phi_{m}$, and the intrinsic viscosity of the system, [η] [6,10].

The maximum packing fraction of the disperse solid phase, $\phi_{m}$, is related to the particle size distribution and their shape [1,6]. Thus, in a system of monodisperse rigid spherical particles, the value of $\phi_{m}$ is approximately equal to 0.648, regardless of the size of the sphere. Under this hypothesis, Hu et al. [27] proposed an equation that can be used as a first approximation to the value of $\phi_{m}$. In polydisperse systems, the value of $\phi_{m}$ increases with particle size variability as the space between them is filled more efficiently ($\phi_{m}$ ≈ 0.744) [6,7].

The intrinsic viscosity, [η], depends on the individual effect of particles and their shape on viscosity [1,6]. It takes a value equal to 2.5 for rigid spherical geometries [6]; when the particles deviate of this shape, [η] must be modified [1,13,28,29,30]. The expression suggested by Pabst et al. [28] can be useful to estimate [η]. Anyway, the correlation between particle shape and [η] is fundamentally complicated because, on the one hand, it is normally assumed that all particles have a similar shape and, on the other hand, the fit of [η] in the Krieger–Dougherty equation to the experimental measurements is subject to statistical and systematic errors [28].

As the aggregates move away from the spherical shape, other values of [η] must be used to simulate the actual shape of the particles. Szecsy [30,31] established a relationship between [η] and the circularity of the particle, defined as the relationship between the area and the perimeter of the particle using digital image processing techniques. It is of utmost interest to analyze what occurs with the various concentrations of solids, especially when their values are near the maximum packing fraction, a point that corresponds to the volumetric fraction in which a strong increase of the dynamic viscosity occurs [10]. In the same manner that occurs with cement paste, the variety of shapes and sizes of granular particles is a real fact that introduces a source of randomness related to aggregates to the system.

When the system is diluted the viscosity depends principally on the concentration of particles. Its value does not vary too much when $\dot{\gamma }$ increases since the particles are not close to each other and the hydrodynamic interactions may be disregarded [6,32]. With high concentration of aggregates, which is what happens in SCC, the hydrodynamic interactions that occur between them as a consequence of the shear applied to the suspension [6] must be taken into account in addition to the distribution of shapes and sizes. The parameters [η] and $\phi_{m}$ of the Krieger–Dougherty equation depend on $\dot{\gamma }$, and its product remains practically constant when the aggregates are deemed rigid spheres (it takes an average value equal to 1.9 [6] or 2 [10,33]). Thus, this energy introduces a source of randomness to the system as occurs with the systems comprised of only cement paste.

### 2.2. Bayesian Approach

The Bayesian networks are used in a multitude of disciplines and practical problems [15,21], in which the analysis and interpretation of data are important in taking decisions [16,17,18,19]. A Bayesian network consists of two elements, (G,P); the first, G, is a directed acyclic graph, that has the variables as nodes, and the links, the direct dependencies among the variables. Due to the directed acyclic graph, the variables are ordered, and each variable has no parents or a set of parents, normally a few, that are the variables on which there is a direct dependency. The acyclic graph permits answering questions such as: has a subset of variables *A* new information on another subset *B*, when a third subset *C* is already known? This is a very interesting and useful property. Once we have the graph, which defines the qualitative information on the network, we need to quantify probabilities, and this is done by the second element, P, which is a set of conditional probabilities, one per variable, that gives the conditional probabilities of the variables conditioned on their parents. From this set we can obtain the joint density by multiplying all of the conditional probabilities, that is, we have:

(2) $$f\left(x_{1},x_{2},\cdots ,x_{n}\right)=\prod_{i=1}^{n}p\left(x_{i}|\prod_{i}\right),$$

where $\prod_{i}$ are the subsets of parents of the variables $x_{i}$. Thus, defining a multivariate density means defining the directed acyclic graph, and defining each of the conditional probabilities, one per variable. However, these conditional probabilities are local, because normally involve a small number of variables, that is, easy to be defined. In Bayesian methods, the parameters of these conditional probabilities are assumed random, and then, they are converted into random variables, and incorporated into the Bayesian network. It is clear that, apart from some particular cases, the calculus of probabilities is complicated and normally involves integrals, that lead to very complex problems that have no analytical solution. One way to avoid these calculations is by using simulations. We simulate a very high number of samples and use the sample of the variable, marginal, or conditional, we are interested in, and we inform the user by means of a very large sample, instead of an analytical expression, that in practice is equivalent. The Markov Chain Monte Carlo methods optimize the sampling procedure using some asymptotic properties of the simulation procedures that reduce the sample size drastically. This is the method that is used in this paper.

The Bayesian methods deal with parametric families of distributions, the parameters of which are considered random variables [19,21]. These models do not choose a model of the initial family of distributions, but rather a linear convex combination of the different models of the selected family. This aspect is very important since it permits increasing the collection of models and enables the sample to indicate which is the most appropriate [18,19].

The Bayesian approach of a probabilistic model consists of the following steps [34]:

- Selection of the likelihood family.
- Selection of the prior distribution of the parameters. It deals with a very important step in the methodology since the results for small samples strongly depend on it. The selection of this prior information may be done in the following manner:
  − Using an imaginary sample. For that, an expert is consulted for the purpose of providing a virtual sample of a certain size as the most representative to their prior knowledge.
  − Using previous non-updated information available in the specialized scientific literature.
  − Based on data obtained in our own experiments.
- Obtaining data from the sample.
- Calculation of the posterior distribution.
- Combining the posterior with the likelihood to obtain the predictive distribution, which is the one we used.

### 2.3. Formulation of the Probabilistic Model and Bayesian Analysis of the Krieger–Dougherty Equation

The proposed objective consists of converting the Krieger–Dougherty equation into a parametric model and performing a Bayesian analysis of it. In the first place, it will deal with the case of homogenous suspensions of cement paste. Below, the same procedure is performed for the case of self-compacting mortar and self-compacting concrete, thereby considering them as heterogeneous suspensions of granular particles within a homogeneous fluid, which is the cement paste.

Using the Krieger–Dougherty Equation (1), dynamic viscosity of cementitious systems can be calculated; two main parameters intervene, $\phi_{m}$ and [η], which are going to be dealt with as random variables in the Bayesian analysis. In order for the proposed model to provide reasonable results, it is necessary to have prior adequate information, which may be obtained from the experimental data or consultation with the scientific literature. The quality of the information is fundamental, especially when sufficient data are not available [34]. The Bayesian network of the model, which is to be created, will take into account the random nature of the average value of the dynamic viscosity as well as the variability of the parameters that intervene in the model.

The transformation of the parametric-deterministic model into a parametric-probabilistic model and the Bayesian analysis has been undertaken in this work using the open-source code, OpenBUGS [35]. It involves a Bayesian inference program that uses Markov Chain Monte Carlo methods and the Gibbs Sampling as a basis (the Gibbs Sampling is a particular case of a simulation algorithm of a Markov Chain). These methods successively simulate the density function that has been proposed, which does not necessarily have to be similar to the posterior density function. Each value generated only depends on the value simulated previously (thus the denomination of Markov Chain). Besides, OpenBUGS permits the production of the graph or diagram of the Bayesian network of the model in question. The program simulates the posterior distribution of the parameters of a model, which requires the definition thereof, the incorporation of a collection of data and beginning values, the latter of which are an important aspect in the analysis of the quality of the simulations performed [18]. Insofar as results, the program provides the graph of the hierarchical dependence structure between variables and parameters, the functions of the posterior density of the parameters, and a collection of statistics of the probabilistic model.

#### 2.3.1. Cement Paste Suspensions

The first step is the definition of the Krieger–Dougherty model [2] in a dimensionless format:

(3) $$\eta^{*}={\left(1\;-\;\frac{\phi_{p}}{\phi_{m\;p}}\right)}^{-{\left[\eta \right]}_{p}\;\phi_{m\;p}}$$

where,

$\eta^{*}=\frac{\eta_{p}}{\eta_{w}}$: non-dimensional dynamic viscosity of the cement paste.
$\eta_{p}$: dynamic viscosity of the cement paste.
$\eta_{w}$: dynamic viscosity of the continuous fluid phase of the suspension (water).
$\phi_{p}$: fraction in volume of the disperse solid phase of the suspension (cementitious materials).
$\phi_{m\;p}$: maximum packing fraction of the particles of the disperse solid phase.
${\left[\eta \right]}_{p}$: intrinsic viscosity of the cement paste.

To perform the conversion of the initial deterministic model into a probabilistic model and perform the Bayesian analysis, the variables $\phi_{m\;p}$ and ${\left[\eta \right]}_{p}$ are considered independent random variables, which belong to a family of uniform probability density functions defined within a domain (minimum and maximum values of the validity interval). In addition, it is assumed that the Krieger–Dougherty equation provides the mean of the values of the random variable, $\eta_{p}$, which follows a normal density function (with mean $\mu^{*}$ and standard deviation σ). The observed residue, $\epsilon^{*}$, also follows a normal density function and σ is to be defined by means of a non-informative uniform density function. Therefore, the syntax with which the model is to be defined in statistical notation is the following:

(4) $$\begin{array}{ccc} \eta^{*} & ∼ & N\;[\mu^{*},\nu ] \end{array}$$

(5) $$\begin{array}{ccc} \mu^{*} & = & {\left(1\;-\;\frac{\phi_{p}}{\phi_{m\;p}}\right)}^{-{\left[\eta \right]}_{p}\;\phi_{m\;p}} \end{array}$$

(6) $$\begin{array}{ccc} \phi_{m\;p} & ∼ & U\;[\phi_{m\;p\;\min},\;\phi_{m\;p\;\max}] \end{array}$$

(7) $$\begin{array}{ccc} {\left[\eta \right]}_{p} & ∼ & U\;\left[{\left[\eta \right]}_{p\;\min},\;{\left[\eta \right]}_{p\;\max}\right] \end{array}$$

(8) $$\begin{array}{ccc} \sigma & ∼ & U\;[\sigma_{\min},\;\sigma_{\max}] \end{array}$$

where ν is an auxiliary variable depending on the standard deviation ($\nu =\sigma^{-2}$).

#### 2.3.2. Self-Compacting Mortar Suspensions

Again, the first step is the definition of the Krieger–Dougherty model [2] for self-compacting mortar suspensions in a dimensionless format. From $\eta_{p}$, and through the application of a micromechanical model, the viscosity of any cementitious paste with granular phases in suspension (self-compacting mortar and self-compacting concrete) can be calculated considering it as a two-phase suspension of particles within a viscous fluid [6,7]. The addition of each solid phase produces an increase of the viscosity of the fluid phase, which can be calculated by successively applying the Krieger–Dougherty equation when including each one of the granular phases. For the case of self-compacting mortar suspensions, the viscous fluid is the cement paste and the fine aggregate constitutes the only solid phase, thus the model can be expressed through [7]:

(9) $$\eta^{⋄}={\left(1\;-\;\frac{\phi_{\mathrm{FA}}}{\phi_{m\;\mathrm{FA}}}\right)}^{-{\left[\eta \right]}_{\mathrm{FA}}\;\phi_{m\;\mathrm{FA}}}\;$$

where,

$\eta^{⋄}=\frac{\eta_{\mathrm{SCM}}}{\eta_{p}}$: non-dimensional dynamic viscosity of self-compacting mortar.
$\eta_{\mathrm{SCM}}$: dynamic viscosity of self-compacting mortar.
$\eta_{p}$: dynamic viscosity of cement paste.
$\phi_{\mathrm{FA}}$: fraction in volume of the granular phase of the suspension (fine aggregate).
$\phi_{m\;\mathrm{FA}}$: maximum packing fraction of particles of the granular phase.
${\left[\eta \right]}_{\mathrm{FA}}$: intrinsic viscosity of the system when adding the granular phase.

As occurred with the cement pastes, each one of the parameters of Equation (9) are considered random variables that follow a uniform density function of probability within a domain of validity (with minimum and maximum values defined for each parameter). Likewise, Equation (9) determines the mean of the dynamic viscosity of self-compacting mortar, which follows a normal density function of probability (average, $\mu^{⋄}$, standard deviation, σ). The residual, $\epsilon^{⋄}$, also belongs to a normal family and comprises a density function of the uniform type. Thereby the syntax of the model in statistical notation is defined in this way:

(10) $$\begin{array}{ccc} \eta^{⋄} & ∼ & N\;[\mu^{⋄},\nu ] \end{array}$$

(11) $$\begin{array}{ccc} \mu^{⋄} & = & {\left(1\;-\;\frac{\phi_{\mathrm{FA}}}{\phi_{m\;\mathrm{FA}}}\right)}^{-{\left[\eta \right]}_{\mathrm{FA}}\;\phi_{m\;\mathrm{FA}}} \end{array}$$

(12) $$\begin{array}{ccc} \phi_{m\;\mathrm{FA}} & ∼ & U\;[\phi_{m\;\mathrm{FA}\;\min},\;\phi_{m\;\mathrm{FA}\;\max}] \end{array}$$

(13) $$\begin{array}{ccc} {\left[\eta \right]}_{\mathrm{FA}} & ∼ & U\;\left[{\left[\eta \right]}_{\mathrm{FA}\;\min},\;{\left[\eta \right]}_{\mathrm{FA}\;\max}\right] \end{array}$$

(14) $$\begin{array}{ccc} \sigma & ∼ & U\;[\sigma_{\min},\;\sigma_{\max}] \end{array}$$

#### 2.3.3. Self-Compacting Concrete Suspensions

Similarly to self-compacting mortar, self-compacting concrete can be considered as a two-phase suspension of granular particles, in which the addition of each solid phase produces an increase of the dynamic viscosity. Again, applying successively the Krieger–Dougherty equation when including each one of the solid phases (fine aggregate and coarse aggregate) the model is defined as follows [7]:

(15) $$\eta^{\circ }={\left(1\;-\;\frac{\phi_{\mathrm{FA}}}{\phi_{m\;\mathrm{FA}}}\right)}^{-{\left[\eta \right]}_{\mathrm{FA}}\;\phi_{m\;\mathrm{FA}}}\;{\left(1\;-\;\frac{\phi_{\mathrm{CA}}}{\phi_{m\;\mathrm{CA}}}\right)}^{-{\left[\eta \right]}_{\mathrm{CA}}\;\phi_{m\;\mathrm{CA}}}$$

where,

$\eta^{\circ }=\frac{\eta_{\mathrm{SCC}}}{\eta_{p}}$: non-dimensional dynamic viscosity of self-compacting concrete.
$\eta_{\mathrm{SCC}}$: dynamic viscosity of self-compacting concrete.
$\eta_{p}$: dynamic viscosity of cement paste.
$\phi_{\mathrm{FA}}$: fraction in volume of the finer granular phase of the suspension (fine aggregate).
$\phi_{m\;\mathrm{FA}}$: maximum packing fraction of particles of the finer granular phase.
${\left[\eta \right]}_{\mathrm{FA}}$: intrinsic viscosity of the system when adding the finer granular phase.
$\phi_{\mathrm{CA}}$: fraction in volume of the thicker granular phase of the suspension (coarse aggregate).
$\phi_{m\;\mathrm{CA}}$: maximum packing fraction of the thicker granular phase of the suspension (coarse aggregate).
${\left[\eta \right]}_{\mathrm{CA}}$: intrinsic viscosity of the system when adding the coarser granular phase.

Similarly to cement pastes and self-compacting mortars, the parameters of the Krieger–Dougherty equation in self-compacting concretes are treated as random variables with a uniform density function of probability within a domain of validity (with minimum and maximum values defined for each parameter). Thus, the Krieger–Dougherty equation gives the mean of the dynamic viscosity of self-compacting concrete, which follows normal density function of probability (average, $\mu^{\circ }$, standard deviation, σ). Again, the residual, $\epsilon^{\circ }$, belongs to a normal family and comprises a density function of the uniform type. The syntax of the model in a statistical format is shown below:

(16) $$\begin{array}{ccc} \eta^{\circ } & ∼ & N\;[\mu^{\circ },\nu ] \end{array}$$

(17) $$\begin{array}{ccc} \mu^{\circ } & = & {\left(1\;-\;\frac{\phi_{\mathrm{FA}}}{\phi_{m\;\mathrm{FA}}}\right)}^{-{\left[\eta \right]}_{\mathrm{FA}}\;\phi_{m\;\mathrm{FA}}}\;{\left(1\;-\;\frac{\phi_{\mathrm{CA}}}{\phi_{m\;\mathrm{CA}}}\right)}^{-{\left[\eta \right]}_{\mathrm{CA}}\;\phi_{m\;\mathrm{CA}}} \end{array}$$

(18) $$\begin{array}{ccc} \phi_{m\;\mathrm{FA}} & ∼ & U\;[\phi_{m\;\mathrm{FA}\;\min},\;\phi_{m\;\mathrm{FA}\;\max}] \end{array}$$

(19) $$\begin{array}{ccc} \phi_{m\;CA} & ∼ & U\;[\phi_{m\;CA\;\min},\;\phi_{m\;CA\;\max}] \end{array}$$

(20) $$\begin{array}{ccc} {\left[\eta \right]}_{\mathrm{FA}} & ∼ & U\;\left[{\left[\eta \right]}_{\mathrm{FA}\;\min},\;{\left[\eta \right]}_{\mathrm{FA}\;\max}\right] \end{array}$$

(21) $$\begin{array}{ccc} {\left[\eta \right]}_{\mathrm{CA}} & ∼ & U\;\left[{\left[\eta \right]}_{\mathrm{CA}\;\min},\;{\left[\eta \right]}_{\mathrm{CA}\;\max}\right] \end{array}$$

(22) $$\begin{array}{ccc} \sigma & ∼ & U\;[\sigma_{\min},\;\sigma_{\max}] \end{array}$$

## 3. Materials and Methods

### 3.1. Cement Paste Suspensions

**Figure 1:**
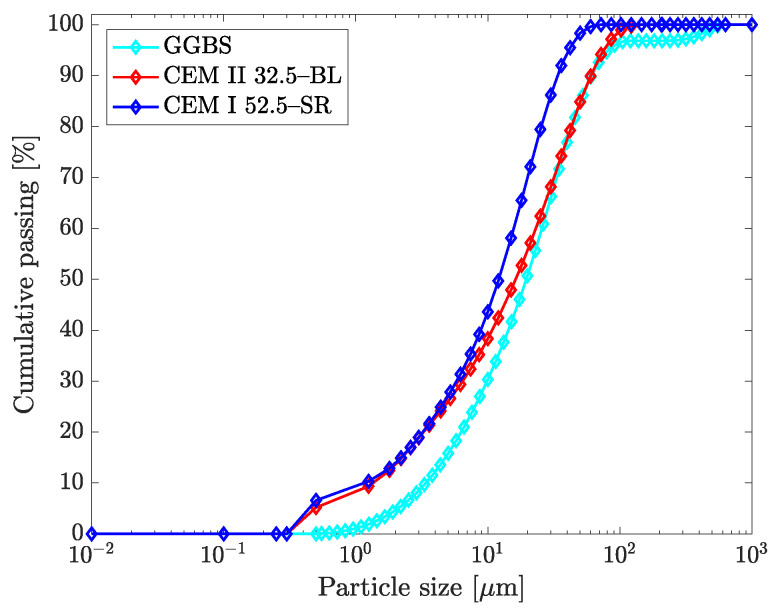
Particle size distribution of the cementitious materials.

The cement pastes analyzed here belong to an experimental campaign recently published by De La Rosa et al. [5]. The cementitious suspensions were elaborated with two classes of Portland cement (*c*) and a mineral addition (ground granulated blast-furnace slag, GGBS). The particle size distribution of the cementitious materials (see Figure 1) was obtained by laser diffraction granulometry using an optical system Mastersizer 2000 (Malvern, UK). Two superplasticizer admixtures of a polymeric nature were used: one based on modified polycarboxylates in an aqueous base with a density equal to 1090 kg/m${}^{3}$ and a dry solid residue of 40% (Sika ViscoCrete-20 HE), and the other of a poly-aryl-ether type with a density equal to 1058 kg/m${}^{3}$ and a dry solid residue of 30% (BASF MasterEase-5025). The composition of the cement pastes in function of the different combinations of materials is the following:

- 31 cement pastes with w/cm relationships equal to 0.35, 0.47, 0.53, and 0.63, each of which was composed of CEM I 52.5-SR, CEM II 32.5 B-L, 75% CEM I 52.5-SR, and 25% GGBS, 75% CEM II 32.5 B-L and 25% GGBS, respectively, and two relationships *SP*/cm equal to 0.4 and 0.8% of a superplasticizer admixture based on polycarboxylates modified in an aqueous based (SikaViscoCrete-20 HE).
- 8 cement pastes with w/cm relationships equal to 0.40, 0.47, 0.53, 0.63, made with CEM I 52.5-SR, and two relationships *SP*/cm equal to 1.0 and 1.2%, with a superplasticizer admixture of a poly-aryl-ether type (BASF MasterEase-5025).

The mixture of the pastes was performed in the following manner: introduction of the cement materials and 90% of the water in the mixer and a 30 s rest. Then, mixing with a rotational velocity of the blades of 64 revolutions per minute (rpm) for 60 s. Subsequently, the mixing stopped, the material stuck to the sides of the recipient, the blades were scraped, and the superplasticizer was added with the remaining water. The mixing was restarted at a velocity of 92 rpm and after 90 s the process was stopped. This mixing protocol was adopted in accordance with the recommendations ASTM C305-99 and AASHTO T162-04 [36,37].

The dynamic viscosity of the cement pastes was measured using a rotational rheometer with a double cone-plate sensor Haake RS50 (Thermo Fischer), performing the tests at a constant temperature (25 ${}^{\circ }$C) with a water bath while controlling the shear rate and preventing the slippage between the suspension and the walls of the sensor. Measurements were performed twice with increasing and decreasing shear rate ramps from 0 to 600 s${}^{-1}$ for 3 min and dwelling time of 1 min at maximum rate. The samples were introduced in the rheometer and a first measurement was taken (considered as a pre-shear test). Subsequently, the sample was at rest for 60 s and then the test was repeated. The procedure was repeated again to have two measurements of $\eta_{p}$ for each sample (78 data).

**Figure 2:**
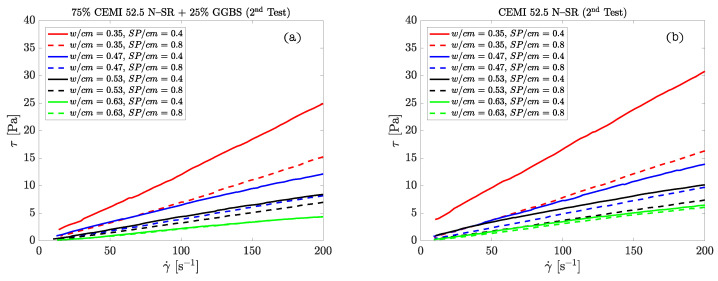
Descending branches of the flow curves of cement pastes obtained with the rotational double cone-plate rheometer: (**a**) 75% CEM I 52.5 N-SR + 25% ground granulated blast-furnace slag (GGBS); (**b**) CEM I 52.5 N-SR.

The resulting flow curves are deemed that formed by the pairs of points shear stress-shear rate of the descent ramp. This permits determining the flow curve of cement suspensions (Figure 2). The slope of the adjusting straight line to the descending branch of the flow curve is $\eta_{p}$, the value of which has been calculated in the range $\dot{\gamma }$ = 10–100 s${}^{-1}$ (note that the linear behavior of the descending branches extends beyond 100 s${}^{-1}$, Figure 2). However, to make correct measurements in the typical range of application of cement pastes for fluid concrete applications (approximately $\dot{\gamma }$ = 100–200 s${}^{-1}$), when preparing the samples it is necessary to subject them to a higher shear rate to obtain a homogeneous and completely deflocculated mixture. Hence, the sample preparation shear rate range reached values of 600 s${}^{-1}$.

The values of the descent branch of the flow curve were adjusted to a Bingham type rheological model in which the value of the slope of the descent branch is $\eta_{p}$. These values of $\eta_{p}$ serve to adjust the parameters $\phi_{m\;p}$ and ${\left[\eta \right]}_{p}$ of the Krieger–Dougherty equation, corresponding to cement pastes. Individual measurements $\eta^{*}$ (= $\eta_{p}/\eta_{w}$) corresponding to each paste are included in Table A1 (Appendix A).

### 3.2. Self-Compacting Mortar/Concrete Suspensions

Next, eight self-compacting mortars from the research of Ouro et al. [38] were analyzed to evaluate the effect of adding the finest portion of a natural sand ($D_{m}$< 1.25 mm) on the parameters of the Krieger–Dougherty equation.

Besides, 17 self-compacting concretes from the research performed by Feys et al. [39], 16 self-compacting concretes from the research performed by Esmaeilkhanian et al. [40], and 9 self-compacting concretes from the doctoral thesis of Grünewald [41] were analyzed. The interest in using these self-compacting concretes lies in the nature and the size of their aggregates: crushed coarse aggregate was used (5–10 mm and 10–20 mm) by Feys et al.; round fine aggregate (0–2 mm) and crushed coarse aggregate (5–10 mm and 5–14 mm) were used by Esmaeilkhanian et al.; Grünewald employed both round fine (0.125–4 mm) and coarse (4–8 mm and 4–16 mm) aggregates.

The rheological measurement of self-compacting mortars was done using a parallel-plates rotational rheometer; every rheological measure of self-compacting concretes was done with rotational coaxial cylinder viscometers. However, none of the cited researches measured the value of the dynamic viscosity of the cement paste, so we have estimated these values from data of the scientific literature [6,7,42,43] according to Ghanbari et al. [6]. The composition of each of the mortars and concretes studied in this article is given in Appendix A.

## 4. Results and Discussion

The transformation of deterministic into probabilistic models was carried out using a Bayesian analysis methodology. For this purpose, the open-source software OpenBUGS was used which applies Markov Chain Monte Carlo and Gibbs Sampling to perform the simulations. In each model a total of 11,000 iterations were carried out to obtain the samples of the variables that can be interpreted as their density functions, which are the parameters of the deterministic models. We used all simulated samples but the first 1000, which belong to the burn-in stage. We did not experience any multimodal posterior density problem, which can appear in some cases, especially of extreme values or reliability analysis [44]. Besides, we did not consider noises since Bayesian methods deal with mixtures of the selected basic models instead of these models themselves, which provide them with more flexibility to reduce noise effects.

### 4.1. Ranges of the Parameters $\phi_{m\;i}$ and ${\left[\eta \right]}_{i}$ to Be Used in the Bayesian Analysis of Cementitious Suspensions

#### 4.1.1. Cement Paste Suspensions

**Figure 3:**
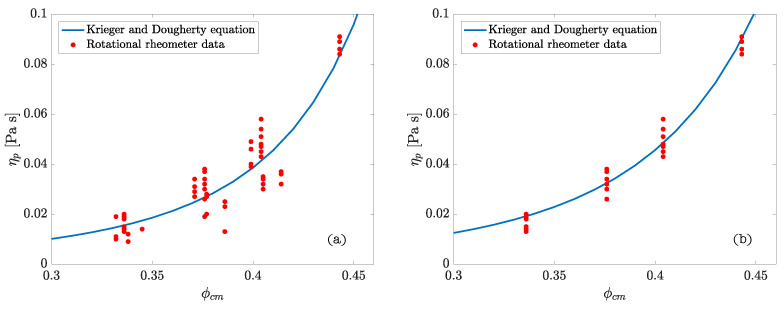
Adjustment curves for the experimental measures to the Krieger–Dougherty equation: (**a**) all the pastes designed; (**b**) CEM I 52.5 N-SR.

From the rheometric tests performed on the cement pastes the values of the parameters $\phi_{m\;p}$ and ${\left[\eta \right]}_{p}$ were calculated by means of the adjustment of the experimental results to the Krieger–Dougherty equation. Some of the adjustment curves may be seen in Figure 3. The results obtained from the statistical adjustment of the data are summarized in Table 1.

Previously, Struble et al. [1], and subsequently, Burgos-Montes et al. [3,45], carried out research on cement pastes of a different nature, that is, pastes with and without mineral additions and superplasticizer admixtures, in which they obtained good results from the adjustment of the experimental data to the Krieger–Dougherty equation. Struble et al. [1] concluded that in cement pastes the parameters $\phi_{m\;p}$ and ${\left[\eta \right]}_{p}$ acquire adjustment values equal to 0.70 and 6.0, respectively. In turn, when the pastes are dispersed with a superplasticizer admixture, the value of $\phi_{m\;p}$ varies between 0.64 and 0.80, while the values of ${\left[\eta \right]}_{p}$ range between 4.5 and 6.0, with average values of 0.70 and 5.0 for $\phi_{m\;p}$ and ${\left[\eta \right]}_{p}$, respectively (Table 2).

In light of the results obtained, it may be verified that the ranges of the values experimentally determined for $\phi_{m\;p}$ and ${\left[\eta \right]}_{p}$ are very similar to those contributed by Struble et al. [1]. The differences that appear, basically in the lower value of the parameter $\phi_{m\;p}$, may be due to the physical–chemical interactions arising between the cement particles as a consequence of the chemical nature of the superplasticizer admixtures used in each case.

#### 4.1.2. Self-Compacting Mortar/Concrete Suspensions

To establish the domains of validity of the parameters of the Krieger–Dougherty equation in each phase of self-compacting mortar/concrete, the next hypotheses have been followed. First, we have as reference the criterium established by Abo-Dhaheer et al. [7], who consider rigid spherical particles for which $\phi_{m}$ increases with the addition of solid phases according to theoretical packing values ($\phi_{m}$ = 0.524 for powder phase, $\phi_{m}$ = 0.63 for powder plus fine aggregate phase, and $\phi_{m}$ = 0.74 for powder plus fine and coarse aggregate phase). Also, according to Hu et al. [27], $\phi_{m}$ could de estimated under the same hypothesis as:

(23) $$\phi_{m}\;=\;1\;-\;0.45\;{\left(\frac{d_{m}}{D_{m}}\right)}^{0.19}$$

where $d_{m}$ and $D_{m}$ are the minimum and maximum dimension of the particles, respectively. Having this into account, we select the lower limit of $\phi_{m}$, equal to 0.55, for every phase of self-compacting concrete (powder, powder plus fine aggregate, powder plus fine and coarse aggregate).

To determine the upper limit of $\phi_{m\;\mathrm{FA}}$, we consider the experimental maximum packing fraction measured by Grünewald for fine round aggregate [41]. Furthermore, the experimental maximum packing fraction measured for both fine and coarse round aggregates is 0.809 (40% fine aggregate, 60% coarse aggregate) [41]. Zentar et al. [46] indicate that $\phi_{m}$ is lower with round aggregates ($\phi_{m}$ = 0.793) than with crushed aggregates ($\phi_{m}$ = 0.901). This is explained by the lubricating effect of the powder and cementitious phases, which is more important with crushed aggregates [46]. Taking into account this, and to establish an adequate value of reference, we take as upper limit of $\phi_{m}$ = 0.894 (packing density of all solid particles). This value has been extracted from the investigation of Kwan et al. [47].

The morphology of the particles in any suspension is mainly controlled for the intrinsic viscosity [46]. Its value is lower for round aggregates than for crushed aggregates because these are closer to the spherical shape ([η] = 2.5). The following equation, proposed by Pabst et al. [28], could be used to estimate [η] knowing the aspect ratio of the particles.

(24) $$\left[\eta \right]\;=\;2.5\;+\;0.123\;{\left(\frac{D_{m}}{d_{m}}\;-\;1\right)}^{0.925}$$

Also, there are other relationships between circularity of particle and intrinsic viscosity [30,31]. As the shape of the set of particles in concrete is broad, we have a lot of uncertainty regarding the maximum and minimum values of [η] that can be acquired in each phase, so we select the same interval in each one of them. Thus, we select a lower limit of [η] = 2.5 and an upper limit [η] = 9, taking into account these references.

Abo-Daheer et al. [7] consider the $\phi_{m}\left[\eta \right]$ product (exponent of the Krieger–Dougherty equation) practically constant and equal on average to 1.9 in every phase, since both parameters have an inverse dependence on the shear rate [6,7]. This hypothesis is very close to other models similar to Krieger–Dougherty, such as that of Quemada (exponent equal to 2) [11]. However, in this parameter product, there is no information about the shape, morphology, particle size distribution, etc., of concrete raw materials.

The ranges of values for the parameters in the mortar phase can be extracted from this analysis. Actually, a lower value of $\phi_{m\;\mathrm{FA}}$ in the mortar phase can be calculated through Equation (23), considering monosize sand particles shape like spheres ($\phi_{m}$ = 0.55). The upper value of $\phi_{m\;\mathrm{FA}}$ is selected according to the experimental maximum packing fraction measured by Grünewald for round fine aggregate with a certain grade of polydispersion [41], which is equal to 0.717. Similarly, we select ${\left[\eta \right]}_{\mathrm{FA}}$ within the 2.5 to 9 interval following the investigations of Choi and Szecsy [30,31].

### 4.2. Bayesian Analysis of the Krieger–Dougherty Equation in Cementitious Suspensions

#### 4.2.1. Cement Paste Suspensions

Table 3 presents the intervals of the values detected for the parameters $\phi_{m\;p}$ and ${\left[\eta \right]}_{p}$, which appear to establish the validity domains in order to carry out the Bayesian analysis of the Krieger–Dougherty equation in cement pastes. The ranges in this work are calculated from the experimental measurements performed in [5].

The description of the model proposed from the Krieger–Dougherty equation in cement pastes and the domains of the definition of the parameters are shown below:

(25) $$\begin{array}{ccc} \eta^{*} & ∼ & N\;[\mu^{*},\nu ] \end{array}$$

(26) $$\begin{array}{ccc} \mu^{*} & = & {\left(1\;-\;\frac{\phi_{p}}{\phi_{m\;p}}\right)}^{-{\left[\eta \right]}_{p}\;\phi_{m\;p}} \end{array}$$

(27) $$\begin{array}{ccc} \phi_{m\;p} & ∼ & U\;[0.510,\;0.830] \end{array}$$

(28) $$\begin{array}{ccc} {\left[\eta \right]}_{p} & ∼ & U\;[4.30,\;6.80] \end{array}$$

(29) $$\begin{array}{ccc} \sigma & ∼ & U\;[0,\;400] \end{array}$$

**Figure 4:**
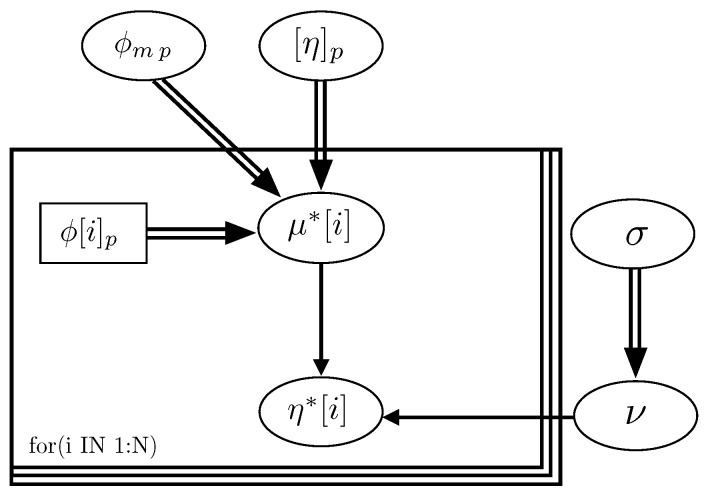
Graph of the Bayesian network of the Krieger–Dougherty equation in cement pastes.

Figure 4 represents the graph of the Bayesian network, obtained for the model, which describes the hierarchical dependence structure of the collection of variables involved. As indicated, the graph of the model permits verifying that $\phi_{m\;p}$, ${\left[\eta \right]}_{p}$ and σ (standard deviation) are independent variables.

**Figure 5:**
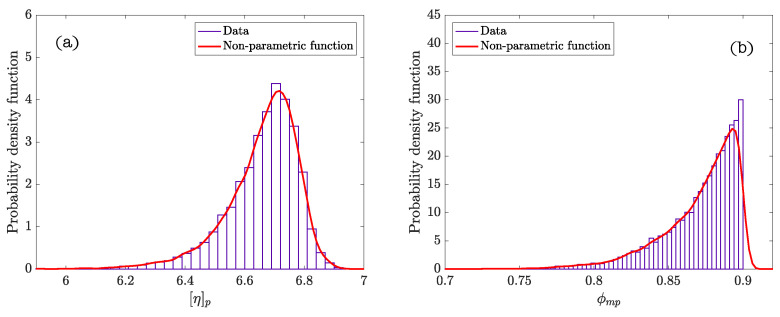
Density functions of probability of the parameters of the cement paste suspensions: (**a**) ${\left[\eta \right]}_{p}$; (**b**) $\phi_{m\;p}$.

**Figure 6:**
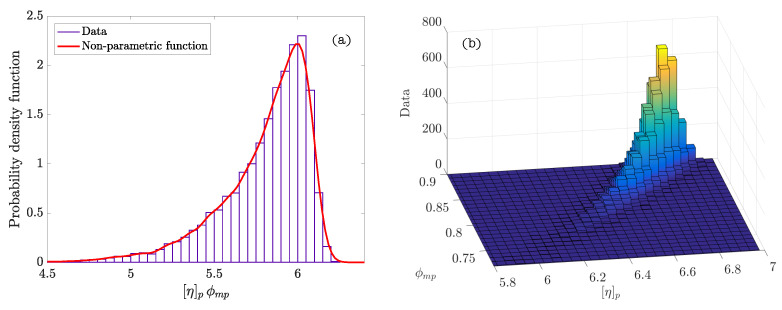
(**a**) Density function of probability of the product of parameters $\phi_{m\;p}\;{\left[\eta \right]}_{p}$ (exponent of the Krieger–Dougherty equation); (**b**) bivariate histogram of $\phi_{m\;p}$ and ${\left[\eta \right]}_{p}$.

Table 4 summarizes the statistics calculated after performing the Bayesian analysis. The graphs of the density functions of the parameters of the Bayesian model are shown in Figure 5. They demonstrate the variability in the distribution of the values of the parameters $\phi_{m\;p}$ and ${\left[\eta \right]}_{p}$, which is indicative of the properties and the nature of these cement systems. Thus, the amplitude of the density function of the parameter $\phi_{m\;p}$ describes the poly-dispersion insofar as the size of the particles within the system. Similarly, the characteristics of the density function of the parameter ${\left[\eta \right]}_{p}$ represents the variability of shapes of the particles present in the suspension. Figure 6a corresponds to the density function of the product of parameters $\phi_{m\;p}\;{\left[\eta \right]}_{p}$ (exponent of the Krieger–Dougherty equation). This function indicates the most likely value that the exponent of the equation in cement pastes can acquire. It reflects what is collected in the scientific literature [1], but now there is a probability density function and not a single value. Figure 6b shows the bivariate histogram of the parameters $\phi_{m\;p}$ and ${\left[\eta \right]}_{p}$.

#### 4.2.2. Self-Compacting Mortar Suspensions

The Krieger–Dougherty model defined for the Bayesian analysis of self-compacting mortars (SCM) from the research of Ouro et al. [38] and the parameter definition domains are as follows:

(30) $$\begin{array}{ccc} \eta^{⋄} & ∼ & N\;[\mu^{⋄},\nu ] \end{array}$$

(31) $$\begin{array}{ccc} \mu^{⋄} & = & {\left(1\;-\;\frac{\phi_{\mathrm{FA}}}{\phi_{m\;\mathrm{FA}}}\right)}^{-{\left[\eta \right]}_{\mathrm{FA}}\;\phi_{m\;\mathrm{FA}}} \end{array}$$

(32) $$\begin{array}{ccc} \phi_{m\;\mathrm{FA}} & ∼ & U\;[0.550,\;0.717] \end{array}$$

(33) $$\begin{array}{ccc} {\left[\eta \right]}_{\mathrm{FA}} & ∼ & U\;[2.5,\;9.0] \end{array}$$

(34) $$\begin{array}{ccc} \sigma & ∼ & U\;[0,\;400] \end{array}$$

**Figure 7:**
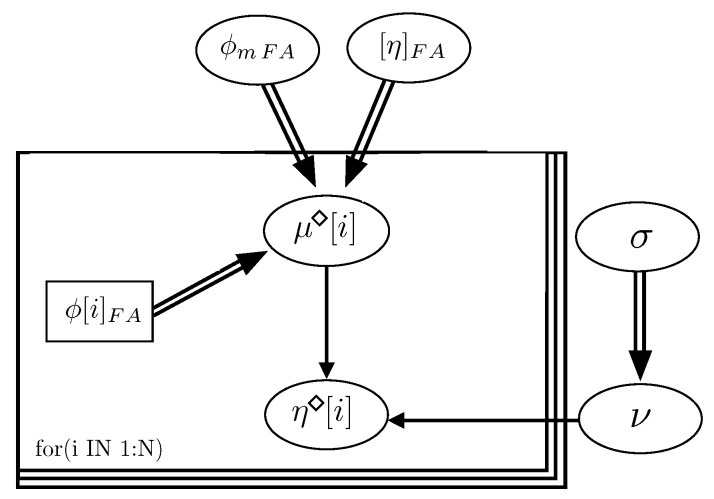
Graph of the Bayesian network of the Krieger–Dougherty equation in self-compacting mortar.

Figure 7 is the graph of the Bayesian network of the model. The statistics calculated from the parameters after performing the analysis are included in Table 5. In this case, mortars were manufactured with the finest portion of a natural sand ($D_{m}$ ≤ 1.25 mm). The shape of the particles is very close to the sphere and we can consider this mortar phase like a monodisperse system, so the calculated values with the Bayesian analysis of the parameters $\phi_{m\;\mathrm{FA}}$ and ${\left[\eta \right]}_{\mathrm{FA}}$ (Table 5) are very close to the theoretical values proposed by Abo-Daheer et al. (which are 0.63 and 2.5, respectively) [7]. Also, the exponent of the Krieger–Dougherty equation $\phi_{m\;\mathrm{FA}}\;{\left[\eta \right]}_{\mathrm{FA}}$ is 1.8, which is very approximate to the theoretical value proposed by Abo-Daheer et al. (which is 1.9) [7].

**Figure 8:**
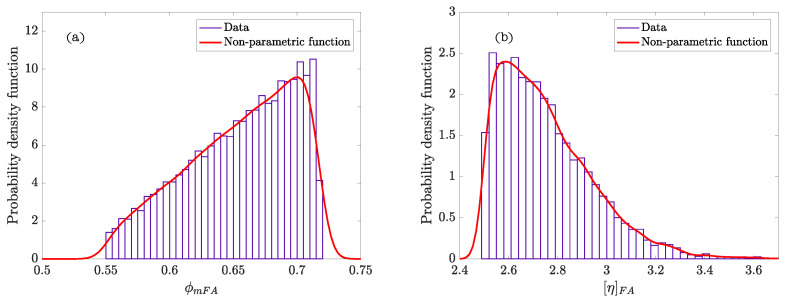
Probability density functions of the parameters of the fine granular phase (FA) in self-compacting mortar: (**a**) $\phi_{m\;\mathrm{FA}}$; (**b**) ${\left[\eta \right]}_{\mathrm{FA}}$.

The graphics of density functions of the parameters are shown in Figure 8. Both $\phi_{m\;\mathrm{FA}}$ and, specially, ${\left[\eta \right]}_{\mathrm{FA}}$ display a very clear peak of probability in a narrow range of values in the mortar. This confirms the hypothesis that the shape of the particles is very close to the sphere and we can consider this mortar phase as a monodisperse system. Thereby, this values are well known in the case of monodisperse system of spheres ($\phi_{m\;\mathrm{FA}}\approx$ 0.63, ${\left[\eta \right]}_{\mathrm{FA}}\approx$ 2.5).

To appreciate the improvement made with the Bayesian analysis better, we can use the mean values of the obtained parameters (Table 5) in the Krieger–Dougherty model to predict the actual experimental data of Ouro et al. [38]. Next, we compare them with the results obtained using the theoretical values proposed by Abo-Daheer et al. ($\phi_{m\;\mathrm{FA}}$ = 0.63 for powder plus fine aggregate phase, $\phi_{m\;i}\;{\left[\eta \right]}_{i}$ = 1.9) [7]. Table 6 shows all the results and the errors obtained with both approaches, expressed as a percentage of the experimental values. The mean error using the theoretical values for the parameters is 43% (standard deviation 39%), which gets reduced to 33% (22%) when the mean values from the Bayesian analysis are used. It should be noted that these errors being high, they are reasonable values according to the uncertainties present in the system and similar to the acceptable error chosen by Ghanbari et al. [6] for their model (25%).

#### 4.2.3. Self-Compacting Concrete Suspensions

The Krieger–Dougherty model defined for the Bayesian analysis of self-compacting concretes (SCC) from the research of Feys et al. [39], Esmaeilkhanian et al. [40], and Grünewald [41]; the parameter definition domains are as follows:

(35) $$\begin{array}{ccc} \eta^{\circ } & ∼ & N\;[\mu^{\circ },\nu ] \end{array}$$

(36) $$\begin{array}{ccc} \mu^{\circ } & = & {\left(1\;-\;\frac{\phi_{\mathrm{FA}}}{\phi_{m\;\mathrm{FA}}}\right)}^{-{\left[\eta \right]}_{\mathrm{FA}}\;\phi_{m\;\mathrm{FA}}}\;{\left(1\;-\;\frac{\phi_{\mathrm{CA}}}{\phi_{m\;\mathrm{CA}}}\right)}^{-{\left[\eta \right]}_{\mathrm{CA}}\;\phi_{m\;\mathrm{CA}}} \end{array}$$

(37) $$\begin{array}{ccc} \phi_{m\;\mathrm{FA}} & ∼ & U\;[0.550,\;0.717] \end{array}$$

(38) $$\begin{array}{ccc} \phi_{m\;\mathrm{CA}} & ∼ & U\;[0.550,\;0.894] \end{array}$$

(39) $$\begin{array}{ccc} {\left[\eta \right]}_{\mathrm{FA}} & ∼ & U\;[2.5,\;9.0] \end{array}$$

(40) $$\begin{array}{ccc} {\left[\eta \right]}_{\mathrm{CA}} & ∼ & U\;[2.5,\;9.0] \end{array}$$

(41) $$\begin{array}{ccc} \sigma & ∼ & U\;[0,\;400] \end{array}$$

**Figure 9:**
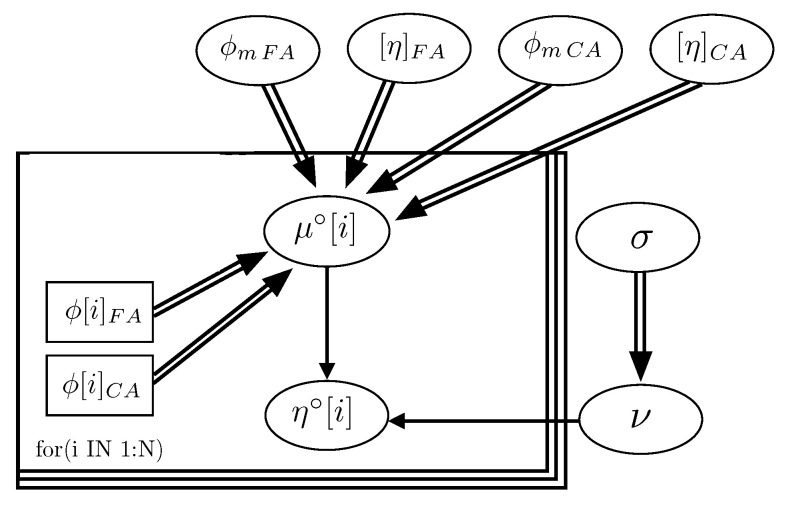
Graph of the Bayesian network of the Krieger–Dougherty equation in self-compacting concretes.

**Figure 10:**
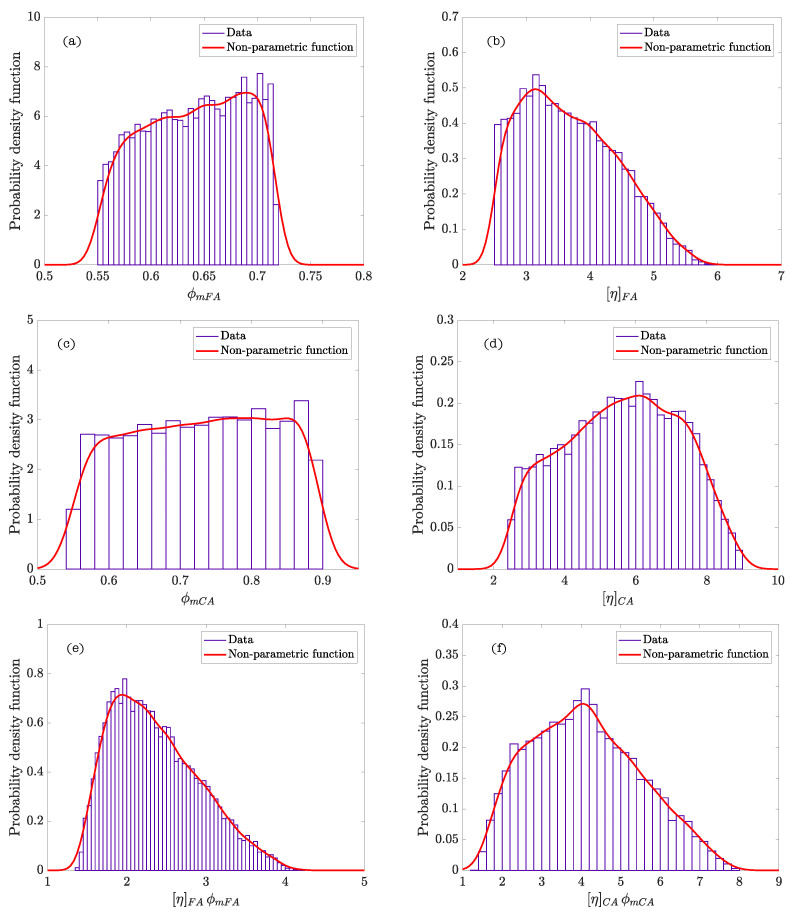
Probability density functions of the parameters of the self-compacting concretes (SCC) phases in Feys et al. [39]: (**a**) $\phi_{m\;\mathrm{FA}}$; (**b**) ${\left[\eta \right]}_{\mathrm{FA}}$; (**c**) $\phi_{m\;\mathrm{CA}}$; (**d**) ${\left[\eta \right]}_{\mathrm{CA}}$; (**e**) ${\left[\eta \right]}_{\mathrm{FA}}\;\phi_{m\;\mathrm{FA}}$; and (**f**) ${\left[\eta \right]}_{\mathrm{CA}}\;\phi_{m\;\mathrm{CA}}$.

**Figure 11:**
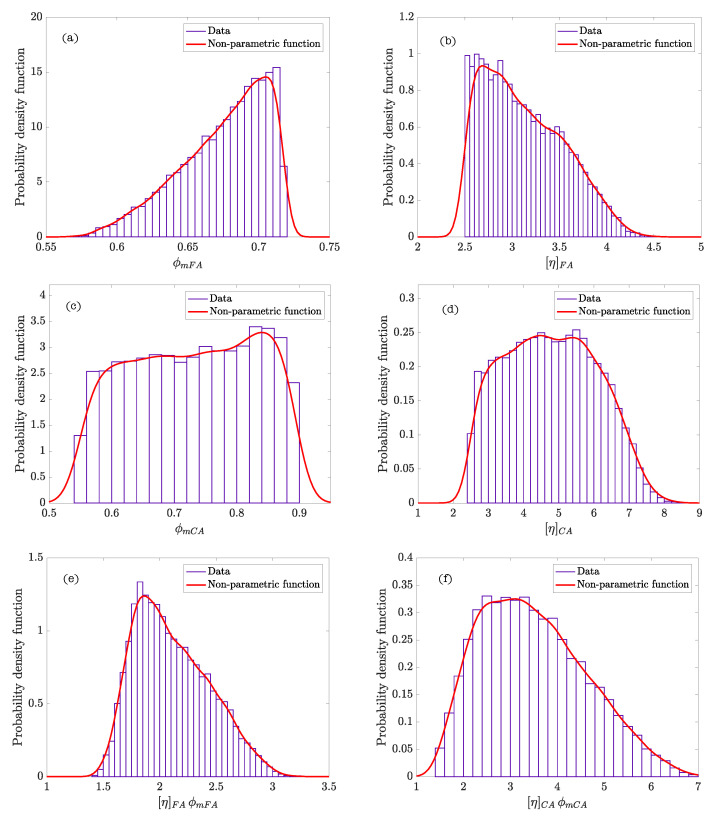
Probability density functions of the parameters of the SCC phases in Esmaeilkhanian et al. [40]: (**a**) $\phi_{m\;\mathrm{FA}}$; (**b**) ${\left[\eta \right]}_{\mathrm{FA}}$; (**c**) $\phi_{m\;\mathrm{CA}}$; (**d**) ${\left[\eta \right]}_{\mathrm{CA}}$; (**e**) ${\left[\eta \right]}_{\mathrm{FA}}\;\phi_{m\;\mathrm{FA}}$; and (**f**) ${\left[\eta \right]}_{\mathrm{CA}}\;\phi_{m\;\mathrm{CA}}$.

**Figure 12:**
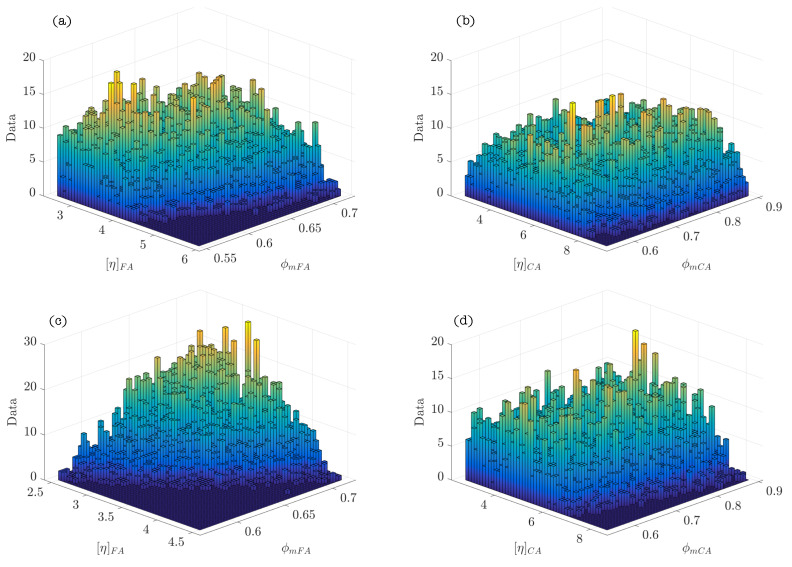
Bivariate histograms in every SCC phase: (**a**) ${\left[\eta \right]}_{\mathrm{FA}}$ and $\phi_{m\;\mathrm{FA}}$ in Feys et al. [39]; (**b**) ${\left[\eta \right]}_{\mathrm{CA}}$ and $\phi_{m\;\mathrm{CA}}$ in Feys et al. [39]; (**c**) ${\left[\eta \right]}_{\mathrm{FA}}$ and $\phi_{m\;\mathrm{FA}}$ in Esmaeilkhanian et al. [40]; and (**d**) ${\left[\eta \right]}_{\mathrm{CA}}$ and $\phi_{m\;\mathrm{CA}}$ in Esmaeilkhanian et al. [40].

The scheme of the Bayesian network of the model, which describes the hierarchy and independence of variables, is represented in Figure 9. The statistics calculated from the parameters of the model after performing the analysis are included in Table 7. The graphics of the functions of density of the parameters of every phase composing SCC and the density function of the product of parameters $\phi_{m\;i}\;{\left[\eta \right]}_{i}$ (exponent of the Krieger–Dougherty equation) are shown in Figure 10 (Feys et al. [39]), and Figure 11 (Esmaeilkhanian et al. [40]). Figure 12 represents the bivariate histogram of the parameters $\phi_{m\;i}$ and ${\left[\eta \right]}_{i}$ in every phase of the research of Feys et al. [39] and Esmaeilkhanian et al. [40].

The sands of the research of Esmaeilkhanian et al. and Grünewald are round aggregates. The mean value of $\phi_{m\;\mathrm{FA}}$ is similar (≈0.68). However, this parameter has a lower value for the sand of Feys et al., which can indicate that is a crushed sand. Regarding ${\left[\eta \right]}_{\mathrm{FA}}$, the mean value calculated for the fine aggregate data of Feys et al. is higher than the value obtained in the data of Esmaeilkhanian et al. This could reinforce the hypothesis of crushed fine aggregate of Feys et al. The value obtained for ${\left[\eta \right]}_{\mathrm{FA}}$ in Grünewald data is the highest (≈4.03). This could indicate that the shape of this fine aggregate has a lower circularity than the other sands [30,31].

The mean value of $\phi_{m\;\mathrm{CA}}$ is similar in the three coarse aggregates (≈0.74). We know that the self-compacting concretes of the research of Feys et al. and Esmaeilkhanian et al. are crushed aggregates whereas the coarse aggregate of Grünewald is rounded. This could be explained by the lubricating effect of the powder and cementitious phases, which is more important with crushed aggregates [46]. Regarding ${\left[\eta \right]}_{\mathrm{CA}}$ the mean values obtained in every research are different and higher than 2.5. This means that the shape of the coarse aggregates moves away from the spherical shape, since the parameter ${\left[\eta \right]}_{\mathrm{CA}}$ is related with the circularity of the aggregate [30,31]. This is plausible for the crushed coarse aggregates employed by Feys et al. and Esmaeilkhanian et al. Also, this is possible for elongated round aggregates. In this particular case, the investigation of Grünewald indicates that sand grains are much more rounded than coarse aggregates [41]. This fact is reflected in the Bayesian analysis of the data as well.

If we look at the non-parametric density functions, $\phi_{m\;\mathrm{FA}}$ displays a peak of probability for the fine aggregate phase of Esmaeilkhanian et al. whereas the density function is smoother for Feys et al. (Figure 10a,b and Figure 11a,b). This fact indicates that the maximum packing of the fine aggregate gets a value with high probability, which could be due to the use of fine round aggregate (the maximum packing value of spheres is well known). The plateau of values with similar probability for the fine aggregate of Feys et al. confirms that crushed fine aggregate was used in this research.

Respect to the coarse aggregate phase, in both cases non-parametric density functions of $\phi_{m\;\mathrm{CA}}$ and ${\left[\eta \right]}_{\mathrm{CA}}$ (Figure 10c,d and Figure 11c,d) show a shape similar to a uniform density function with similar ranges of values and probability. Both investigations use crushed coarse aggregate; this indicates that there exists a variety of shapes of coarse aggregates leading to a significant dispersion in the values of $\phi_{m\;\mathrm{CA}}$.

Figure 10e,f and Figure 11e,f show that the maximum probability of the exponent of the Krieger–Dougherty equation is, approximately, equal to the theoretical value of 1.9 in the fine granular phase. However, the width of the density function in the coarse aggregate phase indicates that the exponent could acquire an ample range of values with similar probability. Figure 12 shows the bivariate histogram of the parameters $\phi_{m\;i}$ and ${\left[\eta \right]}_{i}$ in every phase composing the self-compacting concretes of Esmaeilkhanian et al. and Feys et al.

The improvement made with the Bayesian analysis can be visualized by carrying out the same process that has been done with mortars, applying the mean values of the parameters obtained in the Bayesian analysis (Table 7) of the Krieger–Dougherty model to the data of Feys et al. [39] (see the results in Table 8), Esmaeilkhanian et al. [40] (Table 9), and Grünewald [41] (Table 10), and compare them with the results obtained with the theoretical values proposed by Abo-Daheer et al. [7] ($\phi_{m\;\mathrm{FA}}$ = 0.63 for powder plus fine aggregate phase; $\phi_{m\;\mathrm{CA}}$ = 0.74 for powder plus fine and coarse aggregate phase; $\phi_{m\;i}\;{\left[\eta \right]}_{i}$ = 1.9).

Again, the error given by the Krieger–Dougherty model in calculating the dynamic viscosity of the SCC is smaller in the three series of tests. Namely, the mean error goes from 77% (standard deviation of 8%) to 25% (24%) in the Feys et al. series [39], from 42% (24%) to 36% (26%) in the Esmaeilkhanian et al. tests [40] and from 71% (9%) to only 17% (22%) in the series reported by Grünewald [41]. In this last series of calculations, it is noteworthy that the error of most of the predicted values (8 out of 9) is smaller than 25%, i.e., the predictions are excellent according to the criterion established by Ghanbari et al. [6].

## 5. Conclusions

We carried out the transformation of the Krieger–Dougherty equation into a probabilistic model using a Bayesian analysis methodology. The open-source software OpenBUGS was used, which employs Markov Chain Monte Carlo and Gibbs Sampling to perform the simulations to obtain the samples of the variables that can be interpreted as their density functions, which are the parameters of the deterministic models. The methodology has been applied to cement pastes, self-compacting mortars, and self-compacting concretes. The density functions of the parameters (maximum packing fractions of the phases involved, $\phi_{m\;i}$ and their corresponding intrinsic viscosities ${\left[\eta \right]}_{i}$) are conditioned by the Bayesian graph, which describes the hierarchy and independence of variables, by the limits of the initial uniform distributions and by the limits of the final distribution. The analysis does not attribute the resulting distribution to a single cause (for example, the variations in the shape of the aggregate), but to all those that can physically condition the values of the parameters.

In particular, the Bayesian method applied to the cement pastes in De La Rosa et al. [5] confirms that the theoretical values give reasonable results and, for the first time, calculates the distribution function of the parameters of the Krieger–Dougherty equation. The results obtained with the self-compacting mortars of Ouro et al. [38] also confirm that theoretical values give a good approximation, and the abrupt shape of the distribution function for ${\left[\eta \right]}_{\mathrm{FA}}$ indicates that the used sand was round and spherical. Additionally, the error in the dynamic viscosity predictions using the mean values of the distribution curves (33%) is less than the error with the theoretical values (43%) and the standard deviation is also reduced.

We also applied the Bayesian methodology to three series of self-compacting concretes reported by Feys et al. [39], Esmaeilkhanian et al. [40], and Grünewald [41]. The Bayesian results detect that the sand used by Feys et al. [39] was not round but crushed, since the maximum packing fraction $\phi_{m\;\mathrm{FA}}$ is smaller than in the other cases (0.64 versus 0.68) and the distribution is smoother. Similarly, the methodology reveals that the sand used by Grünewald [41] was less spherical than the others due to the high value obtained for ${\left[\eta \right]}_{\mathrm{FA}}$. The study also reveals that the coarse aggregates used in the three SCC series must have low sphericity due to the high values obtained for ${\left[\eta \right]}_{\mathrm{CA}}$ in all of them. Regarding the exponents of the Krieger–Dougherty equation, the one for fine aggregates is closer to the theoretical value (1.9) for the fine aggregate in the three series, whereas the uniform distribution for the exponents corresponding to the coarse aggregates reveals, again, the disparity in the shape of the crushed particles. Finally, the dynamic viscosity predictions made with the mean values of the Bayesian distributions were significantly better than those made with the theoretical values. The error diminished from 77% to 25% in the Feys et al. [39] series, from 42% to 36% in the Esmaeilkhanian et al. [40] SCCs and from 71% to 17% in the Grünewald [41] concretes.

## Figures and Tables

**Table 1:** Values of $\phi_{m\;p}$ and ${\left[\eta \right]}_{p}$ calculated in cement pastes [5]. Average range in rotational rheometry: 0–600 $s^{-1}$. CI stands for confidence interval.

Cementitious Material	SP/cm	$\eta_{w}$ [mPa s]	$\phi_{m\;p}$	95% CI	${\left[\eta \right]}_{p}$	95% CI	$R_{\mathrm{adj}}^{2}$
CEM I 52.5 N-SR	0.4	0.933	0.68	0.43–0.93	6.6	4.4–7.7	0.980
	0.8	0.933	0.70	0.41–0.98	6.4	3.9–7.4	0.977
	0.4, 0.8	0.933	0.68	0.41–0.96	6.5	4.0–7.6	0.922
	1.0	0.891	0.75	0.56–0.94	6.8	5.5–7.7	0.981
	1.2	0.891	0.69	0.62–0.76	6.5	5.9–6.9	0.995
	1.0, 1.2	0.891	0.72	0.63–0.80	6.7	6.1–7.1	0.986
	0.4, 0.8, 1.0, 1.2	0.911	0.74	0.66–0.83	6.8	6.3–7.2	0.973
CEM II 32.5 BL-II	0.4	0.933	0.57	0.53–0.61	5.0	4.6–5.3	0.999
	0.8	0.933	0.83	0.54–1.00	6.2	4.8–6.8	0.993
	0.4, 0.8	0.933	0.66	0.52–0.79	5.5	4.5–6.2	0.979
75% CEM I 52.5 N-SR + 25% GGBS	0.4	0.933	0.60	0.36–0.84	6.0	2.8–7.3	0.954
	0.8	0.933	0.83	0.50–1.00	6.5	4.8–7.3	0.991
	0.4, 0.8	0.933	0.63	0.41–0.85	6.1	3.9–7.2	0.917
75% CEM II 32.5 BL-II + 25% GGBS	0.4	0.933	0.69	0.26–1.00	6.0	0.2–7.3	0.950
	0.8	0.933	0.51	0.45–0.56	4.3	3.6–4.9	0.987
	0.4, 0.8	0.933	0.58	0.42–0.73	5.1	3.4–6.1	0.909
All	0.4, 0.8, 1.0, 1.2	0.911	0.61	0.55–0.67	5.7	5.2–6.2	0.837

**Table 2:** Values of the parameters $\phi_{m\;p}$ and ${\left[\eta \right]}_{p}$ in cement pastes calculated by Struble et al. [1]. Range of measure in rotational rheometer: 0–600 $s^{-1}$.

Cement Paste	$\dot{\gamma }$ $\left[s^{-1}\right]$	$\phi_{m\;p}$	${\left[\eta \right]}_{p}$
Cement type I (dispersed)	25	0.64	5.1
Cement type I (dispersed)	500	0.76	6.2
Cement type I (floculated)	500	0.64	6.3
White cement (dispersed)	25	0.67	5.7
White cement (dispersed)	500	0.80	6.8
Cement type V (dispersed)	Low limit	0.70	4.7
Cement type V (dispersed)	25	0.68	4.5
Cement type V (dispersed)	500	0.75	5.2

**Table 3:** Definition domains of the parameters $\phi_{m\;p}$ and ${\left[\eta \right]}_{p}$.

Investigation	$\phi_{m\;p}$	${\left[\eta \right]}_{p}$
Struble et al. [1]	0.64–0.80	4.5–6.8
Present work & De La Rosa et al. [5]	0.51–0.83	4.3–6.8

**Table 4:** Statistics of the parameters $\phi_{m\;p}$ and ${\left[\eta \right]}_{p}$.

Parameter	Mean	Std. Dev.	Percentage 2.5%	Median	Percentage 97.5%
$\phi_{m\;p}$	0.870	0.031	0.800	0.879	0.899
${\left[\eta \right]}_{p}$	6.651	0.175	6.333	6.681	6.829

**Table 5:** Statistics of the parameters $\phi_{m\;\mathrm{FA}}$ and ${\left[\eta \right]}_{\mathrm{FA}}$ for self-compacting mortar.

Investigation	Parameter	Mean	Std. Dev.	Percentage 2.5%	Median	Percentage 97.5%
Ouro et al. [38]	$\phi_{m\;\mathrm{FA}}$	0.655	0.043	0.565	0.662	0.715
	${\left[\eta \right]}_{\mathrm{FA}}$	2.753	0.196	2.510	2.714	3.221

**Table 6:** Comparison between experimental values and model results for self-compacting mortar (Ouro et al. [38]).

Name	Experimental $\eta_{\mathrm{SCM}}$ [Pa s]	Calculated with Theoretical Values [7] $\eta_{\mathrm{SCM}}$ [Pa s]	Calculated with Bayesian Mean Values, $\eta_{\mathrm{SCM}}$ [Pa s]	Error with Theoretical Values [7] [%]	Error with Bayesian Mean Values [%]
N3, N4	1.90	1.35	1.17	29.0	38.6
N7, N8	1.10	1.12	0.98	2.2	11.3
N11	1.66	1.48	1.26	10.5	24.1
N13	1.41	1.10	0.97	21.9	31.3
N15	0.94	1.28	1.08	36.0	15.3
E3, E4	0.50	1.06	0.87	112.4	73.3
E7	0.35	0.49	0.40	41.2	15.2
E13	0.37	0.72	0.59	93.3	59.6

**Table 7:** Statistics of the parameters $\phi_{m\;\mathrm{FA}}$, $\phi_{m\;\mathrm{CA}}$, ${\left[\eta \right]}_{\mathrm{FA}}$, and ${\left[\eta \right]}_{\mathrm{CA}}$ for self-compacting concretes.

Investigation	Parameter	Mean	Std. Dev.	Percentage 2.5%	Median	Percentage 97.5%
Feys et al. [39]	$\phi_{m\;\mathrm{FA}}$	0.640	0.047	0.557	0.643	0.713
	${\left[\eta \right]}_{\mathrm{FA}}$	3.682	0.743	2.565	3.597	5.206
	$\phi_{m\;\mathrm{CA}}$	0.728	0.099	0.560	0.731	0.886
	${\left[\eta \right]}_{\mathrm{CA}}$	5.611	1.607	2.713	5.672	8.402
Esmaeilkhanian et al. [40]	$\phi_{m\;\mathrm{FA}}$	0.675	0.031	0.605	0.682	0.716
	${\left[\eta \right]}_{\mathrm{FA}}$	3.120	0.425	2.525	3.052	4.009
	$\phi_{m\;\mathrm{CA}}$	0.730	0.099	0.560	0.734	0.887
	${\left[\eta \right]}_{\mathrm{CA}}$	4.776	1.291	2.628	4.751	7.151
Grünewald [41]	$\phi_{m\;\mathrm{FA}}$	0.682	0.029	0.610	0.689	0.716
	${\left[\eta \right]}_{\mathrm{FA}}$	4.032	0.425	2.954	4.102	4.679
	$\phi_{m\;\mathrm{CA}}$	0.740	0.097	0.562	0.749	0.887
	${\left[\eta \right]}_{\mathrm{CA}}$	5.294	1.094	3.183	5.241	7.741

**Table 8:** Comparison between experimental values and models, and error estimated for self-compacting concrete (Feys et al. [39]).

Name	Experimental $\eta_{\mathrm{SCC}}$ [Pa s]	Calculated with Theoretical Values [7] $\eta_{\mathrm{SCC}}$ [Pa s]	Calculated with Bayesian Mean Values, $\eta_{\mathrm{SCC}}$ [Pa s]	Error with Theoretical Values [7] [%]	Error with Bayesian Mean Values [%]
SCC1	50.8	11.0	50.7	78.3	0.1
SCC2	42.6	11.1	49.8	74.1	17.0
SCC3	38.0	10.9	49.4	71.2	30.0
SCC4	41.4	11.4	52.9	72.5	27.8
SCC7	67.5	12.1	56.8	82.1	15.9
SCC8	59.0	11.5	53.5	80.4	9.3
SCC9	28.0	10.3	47.1	63.2	68.4
SCC10	45.0	10.5	50.1	76.6	11.3
SCC11	35.0	8.1	34.5	76.7	1.4
SCC12	96.5	15.2	78.2	84.2	19.0
SCC13	41.5	11.1	50.3	73.4	21.3
SCC14	29.3	11.5	53.9	60.7	84.1
SCC15	49.6	10.6	48.1	78.6	3.0
SCC16	55.6	10.5	51.9	81.2	6.6
SCC17	44.8	9.8	52.2	78.1	16.6
SCC18	71.2	11.3	52.1	84.1	26.7
SCC19	71.2	12.3	58.1	92.1	62.7

**Table 9:** Comparison between experimental values and models, and error estimated for self-compacting concrete (Esmaeilkhanian et al. [40]).

Name	Experimental $\eta_{\mathrm{SCC}}$ [Pa s]	Calculated with Theoretical Values [7] $\eta_{\mathrm{SCC}}$ [Pa s]	Calculated with Bayesian Mean Values, $\eta_{\mathrm{SCC}}$ [Pa s]	Error with Theoretical Values [7] [%]	Error with Bayesian Mean Values [%]
SCC1	59.3	19.5	34.4	67.2	42.0
SCC2	29.0	17.1	30.1	41.2	3.9
SCC3	69.5	19.6	34.6	71.8	50.2
SCC4	124.0	16.1	28.9	87.0	76.7
SCC5	25.0	17.0	29.9	32.1	19.7
SCC7	62.0	31.6	51.8	49.0	16.5
SCC8	25.0	11.9	21.5	52.2	13.8
SCC9	28.0	32.7	52.6	16.8	87.9
SCC10	72.0	38.0	61.5	47.2	14.6
SCC11	128.0	41.7	67.8	67.4	47.1
SCC12	71.0	37.6	60.8	47.1	14.3
SCC13	67.0	37.4	60.7	44.1	9.4
SCC14	35.0	31.8	51.5	9.0	47.1
SCC16	37.0	31.6	51.2	14.5	38.3
SCC17	39.0	31.6	51.2	18.9	31.2
SCC18	30.0	31.7	51.5	5.7	71.6

**Table 10:** Comparison between experimental values, models, and error estimated for self-compacting concrete (Grünewald [41]).

Name	Experimental $\eta_{\mathrm{SCC}}$ [Pa s]	Calculated with Theoretical Values [7] $\eta_{\mathrm{SCC}}$ [Pa s]	Calculated with Bayesian Mean Values, $\eta_{\mathrm{SCC}}$ [Pa s]	Error with Theoretical Values [7] [%]	Error with Bayesian Mean Values [%]
OS1	69.2	16.1	74.6	76.8	7.9
OS2	59.4	11.4	49.0	80.9	17.4
OS3	87.9	26.0	91.8	70.5	4.5
OS4	56.0	15.8	54.5	71.8	2.7
OS5	97.6	49.3	167.8	49.5	71.9
OS6	81.0	26.3	92.9	67.6	14.6
OS7	62.2	16.2	55.9	74.0	10.1
OS8	71.3	21.1	70.6	70.4	1.0
OS9	57.5	14.1	46.0	75.5	20.0

## Abbreviations

The following abbreviations are used in this manuscript:

CI	Confidence interval
*d*	Diameter of the smallest particle in the system
*D*	Diameter of the largest particle in the system
$d_{m}$	Minimum dimension of granular particle
$D_{m}$	Maximum dimension of granular particle
*G*	Gamma probability function
GGBS	Ground granulated blast-furnace slag
*i*	Number of nodes
*n*	Number of conditional probability densities
*N*	Normal probability function
$p(x_{i}|\pi_{i})$	Probability of $x_{i}$ conditioned to $\pi_{i}$
rpm	Number of revolutions per minute
SCC	Self-compacting concrete
SCM	Self-compacting mortar
SCSFRC	Self-compacting steel–fiber reinforced concrete
*SP*/cm	Superplasticizer–cementitious materials ratio
*U*	Uniform probability function
w/cm	Water–cementitious materials ratio
$\varepsilon^{*}$	Residual error for non-dimensional dynamic viscosity of cement paste
$\varepsilon^{\circ }$	Residual error for non-dimensional dynamic viscosity of self-compacting concrete
$\varepsilon^{⋄}$	Residual error for non-dimensional dynamic viscosity of self-compacting mortar
$\dot{\gamma }$	Shear strain rate
η	Dynamic viscosity
$\eta_{\mathrm{SCC}}$	Dynamic viscosity of self-compacting concrete
$\eta_{\mathrm{SCM}}$	Dynamic viscosity of self-compacting mortar
$\eta_{p}$	Dynamic viscosity of cement paste
$\eta_{w}$	Dynamic viscosity of water
$\eta_{0}$	Dynamic viscosity of the fluid phase in the suspension
$\eta^{*}$	Non-dimensional dynamic viscosity of cement paste
$\eta^{\circ }$	Non-dimensional dynamic viscosity of self-compacting concrete
$\eta^{⋄}$	Non-dimensional dynamic viscosity of self-compacting mortar
[η]	Intrinsic viscosity
${\left[\eta \right]}_{\mathrm{CA}}$	Intrinsic viscosity of the coarse aggregate phase in self-compacting concrete
${\left[\eta \right]}_{\mathrm{FA}}$	Intrinsic viscosity of the fine aggregate phase in self-compacting mortar/concrete
${\left[\eta \right]}_{i}$	Intrinsic viscosity of particles in *i* phase (*i* can be equal to *p* for cement paste,
	*FA* for fine aggregate or *CA* for coarse aggregate)

${\left[\eta \right]}_{p}$	Intrinsic viscosity of cement paste
$\mu^{*}$	Mean value for non-dimensional dynamic viscosity of cement paste
$\mu^{\circ }$	Mean value for non-dimensional dynamic viscosity of self-compacting concrete
$\mu^{⋄}$	Mean value for non-dimensional dynamic viscosity of self– compacting mortar
$\nu =\frac{1}{\sigma^{2}}$	Auxiliary variable for the model of probability
$\pi_{i}$	Set of nodes $X_{i}$ in G
σ	Standard deviation
ϕ	Volume fraction of particles
$\phi_{\mathrm{CA}}$	Volume fraction of coarse aggregate
$\phi_{\mathrm{FA}}$	Volume fraction of fine aggregate
$\phi_{m}$	Maximum packing fraction of particles
$\phi_{m\;\mathrm{CA}}$	Maximum packing fraction of particles in the coarse aggregate phase in SCC
$\phi_{m\;\mathrm{FA}}$	Maximum packing fraction of particles in the fine aggregate phase in SCM/SCC
$\phi_{m\;i}$	Maximum packing fraction of particles in *i* phase (*i* can be equal to *p* for cement
	paste, *FA* for fine aggregate or *CA* for coarse aggregate)
$\phi_{m\;p}$	Maximum packing fraction of particles in cement paste
$\phi_{p}$	Volume fraction of particles in cement paste
G	Acyclic graph directed
P	Associated joint probability density of all nodes
X	Nodes or random variables
τ	Shear stress

## Appendix A. Data Annex

### Appendix A.1. Experimental Dynamic Viscosities of the Cement Pastes

**Table A1:** Experimental dynamic viscosities of the cement pastes.

Paste	$\eta_{p}$ [mPa s]	$\phi_{p}$	*SP* /cm	$\eta^{*}\;$	Paste	$\eta_{p}$ [mPa s]	$\phi_{p}$	*SP* /cm	$\eta^{*}\;$
CEM I 52.5 N-SR	18	0.336	0.4	19.3	
	38	0.376	0.4	40.7	CEM II 32.5 BL-II	36	0.414	0.4	38.6
	51	0.404	0.4	54.7		13	0.386	0.4	13.9
	19	0.336	0.4	20.4		32	0.414	0.8	34.3
	37	0.376	0.4	39.7		23	0.386	0.8	24.7
	58	0.404	0.4	62.2		14	0.345	0.8	15.0
	26	0.376	0.8	27.9		25	0.386	0.8	26.8
	43	0.404	0.8	46.1
	20	0.336	0.8	21.4		46	0.399	0.4	49.3
	30	0.376	0.8	32.2		31	0.371	0.4	33.2
	47	0.404	0.8	50.4		10	0.332	0.4	10.7
	13	0.336	1.0	14.6		49	0.399	0.4	52.5
	32	0.376	1.0	35.9	75% CEM I 52.5 N-SR	34	0.371	0.4	36.4
	47	0.404	1.0	52.8	+ 25% GGBS	40	0.399	0.8	42.9
	84	0.443	1.0	94.3		27	0.371	0.8	28.9
	14	0.336	1.0	15.7		11	0.332	0.8	11.8
	34	0.376	1.0	38.2		39	0.399	0.8	41.8
	54	0.404	1.0	60.6		29	0.371	0.8	31.1
	91	0.443	1.0	102.1
	30	0.376	1.2	33.7		34	0.405	0.4	36.4
	45	0.404	1.2	50.5	75% CEM II 32.5 BL-II	28	0.377	0.4	30.0
	89	0.443	1.2	99.9	+ 25% GGBS	12	0.338	0.4	12.9
	15	0.336	1.2	16.8		35	0.405	0.8	37.5
	48	0.404	1.2	53.9		09	0.338	0.8	9.6
	86	0.443	1.2	96.5					

### Appendix A.2. Dosages and Dynamic Viscosities of the Self-Compacting Mortars

**Table A2:** Dosages and dynamic viscosities of the self-compacting mortars in Ouro et al. [38].

Name	CEM Portland + Fly Ash + Silica Fume [kg/m${}^{3}$]	Water [kg/m${}^{3}$]	SP [kg/m${}^{3}$]	Fine Agg. [kg/m${}^{3}$]	$\eta_{\mathrm{SCM}}$ [Pa s]	$\eta_{p}$ [Pa s]
N3, N4	842	335	7.96	834	1.90	0.331
N7, N8	792	357	7.48	818	1.10	0.286
N11	784	329	7.41	886	1.66	0.313
N13	853	355	8.06	774	1.41	0.313
N15	738	348	6.97	893	0.94	0.268
E3, E4	609	353	6.32	979	0.50	0.169
E7	557	381	5.78	995	0.35	0.079
E13	589	371	6.11	953	0.37	0.124

### Appendix A.3. Dosages and Dynamic Viscosities of the Self-Compacting Concretes

**Table A3:** Dosages and dynamic viscosities of the self-compacting concretes in Feys et al. [39].

Name	CEM Portland + 8% SF (GUbSF)	Water [kg/m${}^{3}$]	SP L + SP S [kg/m${}^{3}$]	Fine Agg. [kg/m${}^{3}$]	Coarse Agg. [kg/m${}^{3}$]	$\eta_{\mathrm{SCC}}$ [Pa s]	$\eta_{p}$ [Pa s]
SCC1	599	185	10.0 + 1.4	863	741	50.8	0.413
SCC2	602	183	10.0 + 1.7	859	726	42.6	0.422
SCC3	605	183	10.0 + 1.5	856	729	38.0	0.422
SCC4	597	186	10.0 + 1.6	871	752	41.4	0.413
SCC7	594	175	10.0 + 2.0	849	737	67.5	0.431
SCC8	645	165	15.3 + 2.4	840	750	59.0	0.458
SCC9	558	197	5.3 + 0.1	857	728	28.0	0.377
SCC10	601	180	10.0 + 1.5	816	760	45.0	0.422
SCC11	644	190	10.0 + 1.4	800	717	35.0	0.422
SCC12	562	171	10.0 + 2.8	879	776	96.5	0.422
SCC13	603	178	10.0 + 1.3	845	724	41.5	0.422
SCC14	598	177	10.0 + 0.0	843	737	29.3	0.422
SCC15	597	182	10.0 + 1.8	838	724	49.6	0.422
SCC16	602	179	10.0 + 1.8	797	789	55.6	0.422
SCC17	596	177	10.0 + 1.6	743	831	44.8	0.422
SCC18	602	179	10.0 + 2.2	851	730	71.2	0.422
SCC19	681	156	20.0 + 4.1	855	782	155.8	0.485

**Table A4:** Dosages and dynamic viscosities of the self-compacting concretes in Esmaeilkhanian et al. [40].

Name	CEM Port. (GU) [kg/m${}^{3}$]	Water [kg/m${}^{3}$]	SF [kg/m${}^{3}$]	SP 1 + SP 2 [kg/m${}^{3}$]	Fly Ash [kg/m${}^{3}$]	Fine Agg. [kg/m${}^{3}$]	Coarse Agg. [kg/m${}^{3}$]	$\eta_{\mathrm{SCC}}$ [Pa s]	$\eta_{p}$ [Pa s]
SCC1	377	165	13	2.55 + 2.15	130	950	715	59.3	0.404
SCC2	344	181	12	1.96 + 1.72	119	950	715	29.0	0.350
SCC3	377	165	13	2.18 + 1.92	130	950	715	69.5	0.404
SCC4	381	165	0	2.45 + 21.5	131	950	715	124.0	0.413
SCC5	320	181	28	2.13 + 1.87	116	950	715	25.0	0.340
SCC7	315	166	11	1.88 + 1.66	109	993	749	62.0	0.350
SCC8	365	191.5	13	2.18 + 1.92	126	917	691	25.0	0.350
SCC9	308	169.5	11	1.92 + 2.02	106	1010	760	28.0	0.331
SCC10	341	153.5	12	1.92 + 2.95	118	1010	760	72.0	0.395
SCC11	374	138	13	3.05 + 3.60	129	1010	760	128.0	0.449
SCC12	341	153.5	12	2.80 + 2.85	118	1010	760	71.0	0.395
SCC13	341	153.5	12	2.90 + 2.95	118	1010	760	67.0	0.395
SCC14	308	169.5	11	2.12 + 1.73	106	1005	756	35.0	0.331
SCC16	308	169.5	11	2.12 + 2.18	106	1005	756	37.0	0.331
SCC17	308	169.5	11	2.12 + 2.18	106	1005	756	39.0	0.331
SCC18	308	169.5	11	2.12 + 2.18	106	1005	759	30.0	0.331

**Table A5:** Dosages and dynamic viscosities of the self-compacting concretes in Grünewald [41].

Name	CEM I 52.5 R [kg/m${}^{3}$]	CEM III 42.5 N [kg/m${}^{3}$]	Water [kg/m${}^{3}$]	SP LR + SP HR [kg/m${}^{3}$]	Fly Ash [kg/m${}^{3}$]	Fine Agg. [kg/m${}^{3}$]	Coarse Agg. [kg/m${}^{3}$]	$\eta_{\mathrm{SCC}}$ [Pa s]	$\eta_{p}$ [Pa s]
OS1	249	155	172	2.58 + 1.58	142	913	682	69.2	0.404
OS2	263	149	181	2.88 + 1.44	173	876	655	59.4	0.413
OS3	249	149	171	2.59 + 2.12	146	1089	508	87.9	0.413
OS4	269	143	181	2.78 + 1.85	173	1045	487	56.0	0.413
OS5	0	335	155	2.10 + 1.26	168	1134	528	97.6	0.413
OS6	0	352	164	2.10 + 1.18	192	1089	508	81.0	0.422
OS7	0	367	173	2.17 + 1.09	217	1045	487	62.2	0.422
OS8	228	151	181	2.68 + 1.49	166	1100	467	71.3	0.395
OS9	246	164	188	2.73 + 1.31	180	1058	449	57.5	0.404
